# Gamma Irradiation-Assisted Synthesis of Cellulose Nanocrystal-Reinforced Gelatin Hydrogels

**DOI:** 10.3390/nano8100749

**Published:** 2018-09-21

**Authors:** Wan Hafizi Wan Ishak, Ishak Ahmad, Suria Ramli, Mohd Cairul Iqbal Mohd Amin

**Affiliations:** 1 Polymer Research Centre (PORCE), School of Chemical Sciences and Food Technology, Faculty of Science and Technology, Universiti Kebangsaan Malaysia, Bangi Selangor 43600, Malaysia; hafizi.wan29@gmail.com (W.H.W.I.); su_ramli@ukm.edu.my (S.R.); 2 Faculty of Pharmacy, University Kebangsaan Malaysia, Jalan Raja Muda Abdul Aziz, Kuala Lumpur 50300, Malaysia; mciamin@ukm.edu.my

**Keywords:** hydrogels, cellulose nanocrystal, gelatin, radiation

## Abstract

Herein, we describe the use of gamma irradiation to prepare hydrogels comprising α-cellulose and cellulose nanocrystal (CNC)-reinforced gelatin in the absence of crosslinking agents. In this study, cellulose was extracted from rice husks by an alkali and bleaching treatment followed by acid hydrolysis to produce CNC. A semi-interpenetrating network (semi-IPN) of hydrogels was developed by the miscibility between gelatin and cellulosic materials. Compared to those prepared from α-cellulose, hydrogels prepared by dispersion of CNCs exhibited remarkably enhanced stiffness and swelling properties, which was ascribed to the uniform distribution of CNCs and their increased crystallinity. Improved pore structure, arrangement, and rigidity of CNC-reinforced gelatin hydrogels, which induced the swelling mechanism resulting in higher and faster water uptake was observed with a scanning electron microscope (SEM), compared to cellulose-reinforced gelatin hydrogels. Moreover, in vitro drug profiling demonstrated that CNC/gelatin hydrogels exhibit good drug loading/release behavior and are thus suitable for use in drug-delivery applications.

## 1. Introduction

Gelatin is a nontoxic, biodegradable, cheap, and nonimmunogenic biopolymer with promising pharmaceutical and medicinal applications. Being a derivative of (naturally occurring) collagen, gelatin exhibits superb biocompatibility and thus attracts increased attention as a material suitable for potential biomedical applications, as exemplified by the recently described gelatin-based hydrogels [1]. 

However, gelatin suffers from certain drawbacks, e.g., it cannot withstand the normal body temperature of 37 °C due to undergoing a sol-gel transition under these conditions, which limits its biological and medical applications [2]. Moreover, gelatin exhibits low in vivo mechanical strength and elasticity as a result of swelling, which, nonetheless, can be mitigated by crosslinking to improve both thermal and mechanical stabilities [3]. 

Among the variety of available crosslinking methods (physical, chemical, and radiation-nduced) [4], chemical crosslinking, based on the introduction of chemical additives such as glutaraldehyde and formaldehyde, is the one most commonly used. However, the presence of such chemical residues in a gelatin structure results in cytotoxicity [5], which makes chemically crosslinked gelatin unsuitable for biomedical and pharmaceutical applications. 

In view of the above, radiation-induced crosslinking appears to be the best possible method due to not involving the use of any chemical reagents or additives. Specifically, gamma radiation is well known to induce gelatin crosslinking [5] and afford a three-dimensional network by forming chemical bonds between molecular backbones [6]. Advantages of radiation-induced polymer degradation include its ability to promote reproducible and quantitative changes without the introduction of chemical reagents and concomitantly occurring product sterilization [7]. In addition to crosslinking, the incorporation of natural fibers (such as cellulose) into gelatin helps to improve the mechanical and thermal properties of the corresponding hydrogels. Generally, radicals are formed from chain scission of the polymer chain in high-energy crosslinking, especially when irradiating dried gelatin or cellulose in the presence of oxygen [8]. The degradation of both polymers credited by the chain scission does not occur without an oxygen environment during irradiation, particularly when irradiating in solution. Prior to crosslinking, the restriction of oxygen and the presence of water also result in the termination of radicals, reflecting both gelatin and cellulosic material, respectively [8,9]. Lepore et al. [10] reported that the amorphous region of cellulose easily trapped water molecules, while water was present in the crystalline region. In this study, the irradiation process was done with the restriction of oxygen and the presence of water to terminate radicals and promote the crosslinking process. Similar procedures have been used for different polymers to produce hydrogels [5,8]. 

Cellulose is one of the best known naturally occurring polysaccharides, being the most earth-abundant polymer and exhibiting advantages of renewability, biodegradability, and nontoxicity [11]. The unique semicrystalline nature of cellulose allows its fibers to be reduced in size and form cellulose nanocrystals (CNCs) upon partial acid hydrolysis. Under controlled conditions, the acid-catalyzed cleavage of cellulose fibers occurs at amorphous regions, leaving highly crystalline segments intact, which results in the production of rodlike nanometer-scale particles (also known as whiskers). Currently, increased attention has been directed at the preparation and applications of nanocellulose as a polymer-reinforcing material [12].

Over the past decade, the use of biopolymer hydrogels produced from synthetic or natural polymers for biomedical and food applications has experienced tremendous popularity increase and has become a hotspot of interest for many research groups worldwide, with notable examples including wound dressing [13], drug delivery [14], and tissue-engineering scaffolds [15]. Herein, we developed a novel green technology of preparing CNC- and original cellulose fiber-reinforced gelatin hydrogels utilizing gamma radiation to determine the effect of cellulosic particle size on hydrogel performance, hypothesizing that the presence of variable-size cellulosic fibers (i.e., micro- and nanocellulose) alters the water-absorption capacity and other properties of these hydrogels. Moreover, incorporation of CNCs was expected to increase the rigidity of the soft-hydrogel network and improve its water-uptake ability, making the thus-obtained crosslinked hydrogels useful for future biomedical applications.

## 2. Materials and Methods 

### 2.1. Materials

Pharmaceutical-grade gelatin was purchased from Halagel (M) Sdn. Bhd. (Kuala Lumpur, Malaysia); sulfuric acid and rice-husk fibers were obtained from Sigma-Aldrich (Kuala Lumpur, Malaysia) and Bernas Sdn. Bhd (Shah Alam, Selangor, Malaysia), respectively.

### 2.2. Preparation of Cellulose Fibers and Cellulose Nanocrystals (CNCs) 

CNCs were extracted from rice husks and characterized as described elsewhere [16]. Raw rice-husk fiber was ground to obtain rice husk powder (125–150 ± 5.5 μm), which was subsequently stirred in 4 wt% aqueous NaOH for 2 h at 80 °C using a mechanical stirrer. The separated fibers were filtered and washed with distilled water to remove alkali-soluble components, and residual lignin in the alkali-treated powder was removed by bleaching, which was performed by 4 h stirring in acetate buffer/2.7 wt% NaClO_2_/distilled water at 80 °C. After sixfold repetition of the bleaching treatment, the remaining cellulose fibers were recovered as described above. CNCs were prepared by 30 min acid hydrolysis of cellulose fibers in 65 wt% H_2_SO_4_ at 50 °C under continuous stirring. The resulting suspension was diluted with cold distilled water to stop the reaction and centrifuged for 10 min at 10,000× *g* rpm. The obtained reaction mixture was dialyzed against distilled water until constant pH, and the thus-produced suspensions were freeze-dried to produce CNC powder.

### 2.3. Hydrogel Fabrication

Three hydrogels were prepared, namely, gelatin hydrogel (100% gelatin), hydrogel A (cellulose/gelatin), and hydrogel B (CNCs/gelatin). Cellulose and CNCs (4%) were separately mixed with distilled water, and the resulting mixtures were homogenized (IKA T-25 homogenizer, IKA-Werke GmbH, Staufen im Breisgau, Germany) for 5 min at 8000 rpm and ultrasonicated (Bransonic CPXH, Branson Ultrasonic, Danbury, CT, USA) for 10 min to obtain homogeneous dispersions. Subsequently, these dispersions were treated with gelatin (96%) and stirred with a magnetic stirrer at 55 °C until homogeneity. The thus-prepared mixtures were exposed to gamma radiation (Gamma Cell 220 Excel, MDS Nordion, Ottawa, ON, Canada) at a dose of 30 kGy (irradiation dose was carried out in accordance with the Food and Drug Administration [17]). Samples were irradiated at 25 °C inside sealed, airtight bags to prevent the presence of oxygen during irradiation.

### 2.4. Morphological Studies

CNC morphology was investigated by transmission electron microscopy (TEM) imaging (Philips CM 30, North Billerica, MA, USA). Contrast enhancement was achieved by staining nanocrystals with 2 wt% aqueous uranyl acetate solution for 1 min. Cellulose was placed on an Al stub and incubated in an oven at 60 °C, and the surface morphology of the thus-obtained samples was observed by scanning electron microscopy (SEM; Philips XL 30, North Billerica, MA, USA). Hydrogels were shock-frozen with liquid nitrogen and freeze-dried, with hydrogel morphology subsequently observed using variable-pressure SEM (VP Leo 1450; 500×, 15 kV, Oberkochen, Germany).

### 2.5. X-ray Diffraction (XRD) Analysis

CNC and cellulose fibers in the form of milled powders were subjected to room-temperature XRD analysis (D8-Advance Bruker AXS GmbH, Oestliche, Rheinbrueckenstr, Karlsruhe, Germany; Cu *K*_α_ radiation (*λ* = 0.1539 nm); 2*θ* = 5–80°), and their crystallinity indices (CrI) were determined by Diffrac. Suite EVA 1.4 software 

### 2.6. Fourier Transform Infrared (FTIR) Spectroscopy

FTIR spectra were recorded in attenuated total reflectance (ATR) mode at room temperature in the range of 4000–500 cm^–1^ on a model 2000 Perkin Elmer instrument (Hopkinton, MA, USA) equipped with a diamond ATR crystal. For characterization, samples were cut into 1 cm × 1 cm specimens (2 mm thick) and placed on the ATR plate.

### 2.7. Rheological Characterization

Hydrogels were prepared by immersion into distilled water at room temperature 2 days before analysis, and their rheological properties were characterized using a rheometer (Anton Paar Physica MRC 301, Anton Paar GmbH, Graz, Austria) with a 14 mm diameter flat plate. Storage moduli (*G*’) were determined at constant temperature (25 °C) and 0.05% shear strain for frequencies of 0.1–10 Hz.

### 2.8. Swelling Degrees

The swelling degrees of hydrogels were determined by evaluating their ability to swell in distilled water at room temperature (25 °C) for 48 h. The samples were dried before being dissolved in distilled water.

(1) $$\mathrm{Swelling}\;\left(\%\right)\;=\;\frac{G_{s}-G_{d}}{G_{d}}\times 100,$$

where *G*_s_ is the sample weight after 48 h swelling in distilled water, and *G*_d_ is the sample weight before immersion.

### 2.9. In Vitro Drug Profiling

A separation-diffusion method [18] was used to entrap riboflavin (model drug) into hydrogels. To determine drug-loading efficiency, disc-shaped hydrogel samples were immersed into 10 mg/mL of riboflavin solution for 24 h, washed with 10 mL of distilled water, and dried in an oven for 24 h at 40 °C. The drug concentration in the residual solution was analyzed by UV-vis spectrophotometry (UV-1800, Shimadzu, Kyoto, Japan) at a wavelength of 445 nm, and drug-loading efficiencies (DL%) were calculated as

(2) $$\mathrm{DL}\;\left(\%\right)\;=\;\frac{W_{\mathrm{dg}}}{W_{g}}\times 100,$$

where *W*_dg_ is the amount of drug loaded into the hydrogel, and *W*_g_ is the initial drug amount.

Drug-release profiles were obtained by immersing drug-loaded hydrogels into 50 mL of simulated intestinal fluid (SIF; pH 7) at 37 °C for 24 h under continuous stirring. At each predetermined time interval, 2 mL of this solution was removed for analysis. The drug solution was placed in a locked tube before it was determined by UV-vis spectrophotometry at the maximum absorbance wavelength of riboflavin (445 nm). For every removed aliquot, 2 mL of fresh SIF was added to maintain the volume of the remaining solution. 

## 3. Results

### 3.1. Morphological Analysis

**Figure 1:**
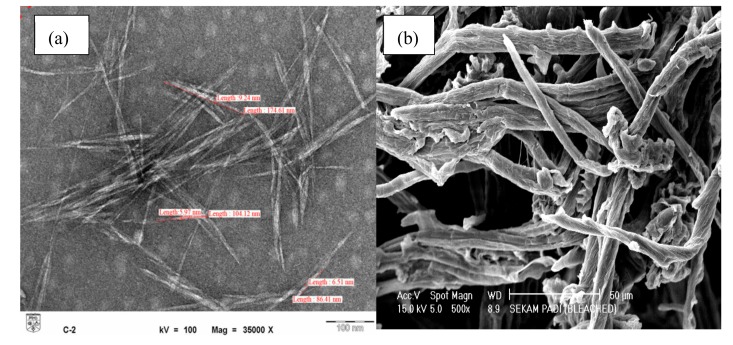
(**a**) Transmission electron microscopy (TEM) image of cellulose nanocrystals (CNCs) and (**b**) scanning electron microscopy (SEM) image of microcellulose fibers.

Sample morphology was analyzed by TEM as previously described for nanomaterial [19]. Figure 1 shows that upon acid hydrolysis, the diameter of individual cellulose fibrils was reduced from 4–8 ± 1.32 μm (pristine fibers) to 5–15 ± 2.77 nm (CNCs), as previously reported [20]. The formation of crystalline rodlike or needlelike particles after partial acid hydrolysis was ascribed to the cleavage of the amorphous region of cellulosic microfibrils [11].

Each individual fibril of cellulose size was reduced from micrometer (Figure 1b) into nanometer (Figure 1a) range after acid hydrolysis treatment. The efficiency of acid hydrolysis treatment was demonstrated by the TEM micrograph (Figure 1a) where a needlelike structure of individual fibers with nanometer size was obtained. Similar results were also reported by Johar et al. [20]. A crystalline rodlike or needlelike particle remained after partial acid hydrolysis due to the cleavage of the amorphous region of cellulosic microfibrils [11]. The obtained CNCs had an aspect ratio of 10–25, with 80% of them having an aspect ratio of 10–20. Thus, the produced CNCs could be well-dispersed in the polymer matrix due to their nanoscale size and thus facilitate the formation of stable-dimension pores in hydrogels to increase their water-absorption capacity [21]. Moreover, the nanoscale size of CNCs was also expected to provide superior surface area for the reaction to occur.

### 3.2. XRD Analysis

**Figure 2:**
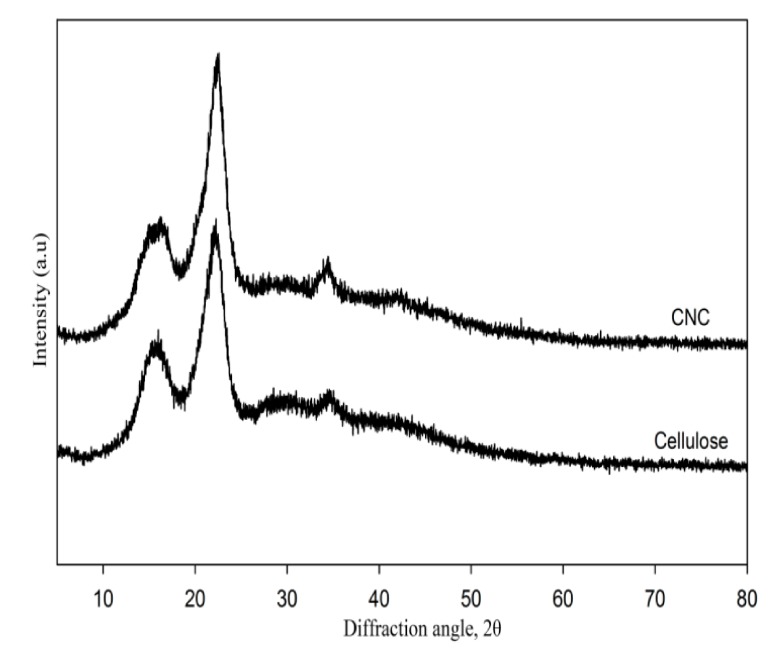
X-ray diffraction patterns of cellulose and CNCs.

Chemical treatment of semicrystalline cellulose (e.g., strong acid-catalyzed hydrolysis) is well-known to influence fiber crystallinity due to inducing the formation of nanocrystals by removal of amorphous parts while keeping the crystalline region intact [22,23]. Herein, differences between the crystallinity of cellulose and CNCs were determined by XRD analysis (Figure 2).

The three well-defined crystalline peaks observed around 2*θ* = 16°, 22°, and 35° were typical of cellulose I, being more intense and sharp for CNCs than for cellulose fiber and thus indicating the success of acid hydrolysis treatment [20]. Thus, hybridization of gelatin-based hydrogels with CNCs was expected to result in stiffness and rigidity, exceeding those obtained in the case of cellulose, i.e., the use of CNCs as reinforcing materials was assumed to increase the mechanical stability of the hydrogel itself. The crystallinity index (CrI) values for all peaks are summarized in Table 1. 

### 3.3. FTIR Analysis

**Figure 3:**
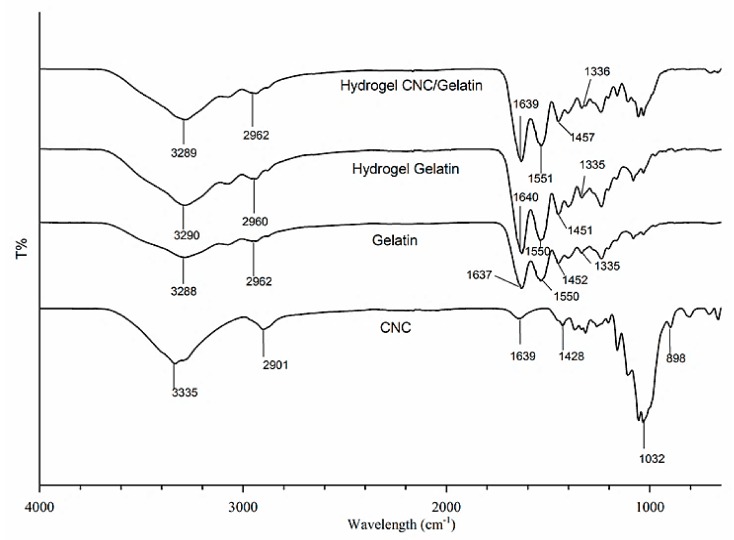
Fourier transform infrared (FTIR) spectra of CNCs, gelatin, and gelatin-based hydrogels.

FTIR characterization was carried out to confirm the crosslinking of gelatin and determine the cellulose reinforcement-induced change of functional groups therein (Figure 3). The CNC spectrum showed absorption peaks at 3335, 1060–1050, and 898 cm^–1^ corresponding to O–H, C–H, and C–O stretches, respectively, in agreement with the results of a previous study [24]. No significant differences were observed between the spectra of original cellulose and CNCs, as reported by Johar et al. [20]; hence, only the spectrum of CNCs is presented herein.

The FTIR spectrum of gelatin showed absorption peaks at 3288 and 2962 cm^–1^, which were assigned to N–H and aliphatic C–H stretches, respectively. Moreover, peaks at 1637 and 1550 cm^–1^ were ascribed to amide C=O stretching (amide I) and amide N–H bending (amide II), respectively, in agreement with previous reports [25]. Finally, absorption peaks at around 1452, 1335, and 1240 cm^–1^ were assigned to C–H bending, C–N stretching, and N–H bending (amide III), respectively, as reported by Rokhade et al. [26].

**Figure 4:**
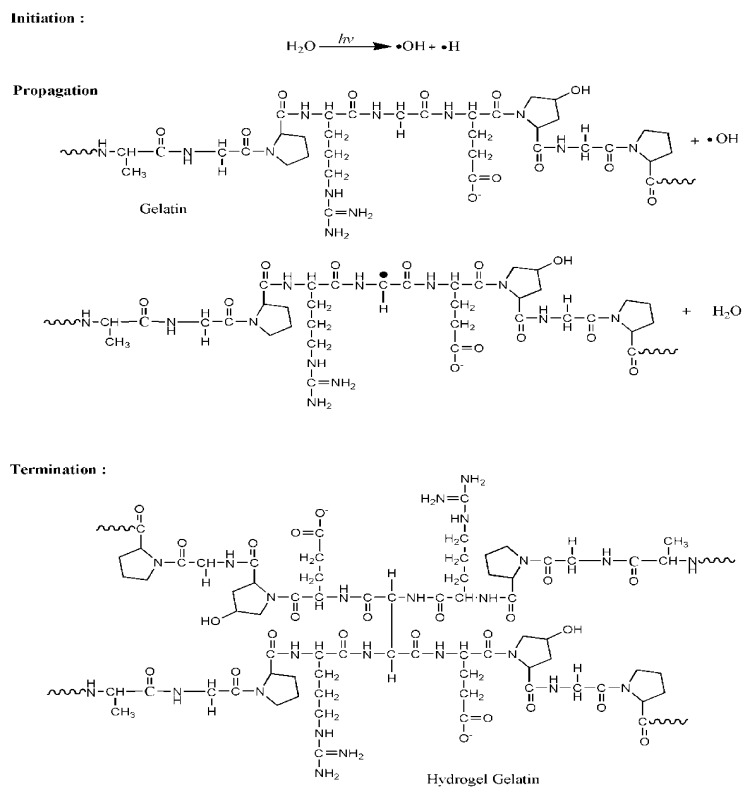
Proposed gelatin-crosslinking mechanism for the formation of hydrogel gelatin by gamma radiation.

Notably, the spectra of gelatin-based hydrogels featured more intense absorptions than those of pure gelatin, which was attributed to gamma irradiation-induced crosslinking, particularly in the formation of hydrogen bonds between the amino acid residues of polymer chains [3]. Generally, absorption peaks located at ~3290 and 1550 cm^–1^ correspond to a triple-helix structure, with their position shift and intensity reduction indicating the breakdown of such structures [8]. On the contrary, crosslinking resulted in an intensity increase of these bands, possibly due to promoting the formation of larger numbers of triple helices. The absorption peaks around 1637 cm^–1^ also gained intensity as a result of crosslinking, which was ascribed to the increased organization of the helical configuration (as compared to random configuration in the absence of crosslinking). The proposed mechanism for gelatin crosslinking is shown in Figure 4. Gamma-radiation energy is mostly absorbed by water in aqueous solutions; hence, water radiolysis mainly yields reactive species, such as proton radicals (H) and hydroxyl radicals (OH). The most reactive species is conceived by OH, which easily remove H in the polypeptide chain, inducing the formation of gelatin radicals and H_2_O as shown in the propagation step. Gelatin radicals are recombined in the termination step by forming covalent bond between the polypeptide chains. The triple helical structure of gelatin was stabilized by the hydrogen bonds formed between peptide bonds in adjacent chains [5].

The absence of significant differences between the spectra of cellulose- and CNC-containing hydrogels implied that both gelatin and cellulose maintained their individual chemical structures therein, with cellulose/CNCs existing in the form of a semi-IPN (interpenetrating polymer network) [12]. Therefore, the excellent miscibility of cellulose and gelatin allowed the formation of a uniform semi-IPN hydrogel network after gamma irradiation-induced crosslinking [5].

### 3.4. Rheological Properties

**Figure 5:**
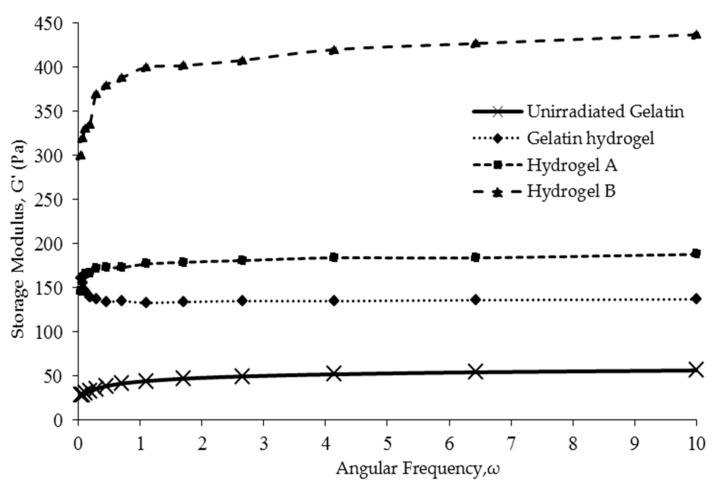
Storage moduli of unirradiated gelatin, gelatin hydrogels, hydrogels A, and hydrogels B.

The dynamic mechanical properties of hydrogels can be characterized in terms of storage modulus (*G*’), which is a measure of material elasticity. Figure 5 shows that the frequency-dependent *G*’ values of hydrogels A and B generally exceeded those of pure-gelatin hydrogel and unirradiated gelatin, which was ascribed to the reinforcing effect of cellulosic fibers. According to Ooi et al. [12], the stiffness of hydrogels increases with an increasing *G*’, which makes them more solid-like and, hence, results in better dynamic mechanical properties.

Figure 5 shows that hydrogels B had the highest storage modulus compared to hydrogels A followed by gelatin hydrogels and unirradiated gelatin. Hydrogels B showed a ~200%–250% higher storage modulus than hydrogel A, which reflected the superiority of the more crystalline CNCs over regular cellulose fibers as reinforcing agents. These results indicate that the higher crystallinity of CNC (Figure 2) had successfully improved hydrogel rigidity and stiffness [12]. Thus, the dynamic mechanical properties of hydrogels might be improved. Similarly, CNCs with a Young’s modulus of over 100 GPa and a surface area of several hundred m^2^ g^−1^ have been reported to exhibit impressive mechanical properties and reinforcing capability [11], allowing the preparation of hydrogels perfectly suited for biomedical applications such as drug delivery, wound dressing, and tissue engineering. 

However, the incorporation of cellulose microfibrils as reinforcing material in hydrogels B also showed a slight increase on the storage modulus compared to neat hydrogels gelatin. The semicrystalline properties of cellulose itself help as supporting components on the gelatin matrix; hence, a more solid-like gel is formed. As for crosslinking, the gamma-radiation technique surely played its role in inducing crosslinking on gelatin. The storage modulus for unirradiated gelatin is just around 50 Pa compared to gelatin hydrogel (radiated), which is about 140 Pa. The significant difference of the storage modulus results from the entanglement of the polypeptide chain due to crosslinking [6]. As crosslinking prevailed, the formation of ordered gelatin helical structures increase, hence reducing random coil formation. These ordered configurations hindered the movement of polymer chains, which remarkably improves dynamic mechanical properties and, hence, produces stiffer gelatin hydrogel. 

### 3.5. Swelling Ratio Test

**Figure 6:**
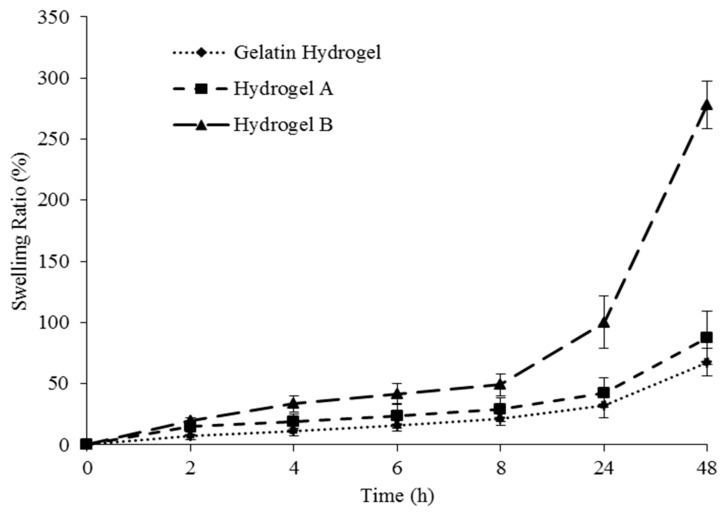
Swelling behavior of different hydrogels.

Figure 6 shows the swelling behavior of gelatin hydrogels reinforced with cellulose (hydrogel A) and CNCs (hydrogel B), with a filler-free gelatin hydrogel used as a control. During swelling, the diffusion of water molecules into the polymer chain induced the formation of a rubbery (swollen) polymer region by facilitating the relaxation of the polymer network [27]. 

The swelling ratio of all hydrogels increased with time and was lowest for the cellulose-free hydrogel, increasing in the case of cellulose- and CNC-containing hydrogels due to the hydrophilicity of the corresponding fillers. Moreover, cellulosic fibers acted as reinforcing materials and stabilized hydrogel pores during polymer-network relaxation caused by the diffusion of water molecules into hydrogels.

Hydrogel B achieved a higher swelling ratio than hydrogel A, with the superior water uptake and water-absorption rate of the former ascribed to the smaller and, hence, better-dispersed cellulosic fibers (CNCs) contained therein, facilitating the formation of rigid and stable pores. According to Razmjou et al. [27], the small particle size of CNCs also provides a greater surface area and interstitial volume, allowing the corresponding hydrogels to hold more water and thus resulting in a higher degree of swelling. In addition, the lower swelling degree observed for hydrogel A can be related to the agglomeration of larger cellulose fibers, which weakens hydrogel uniformity. 

### 3.6. Scanning Electron Microscope (SEM) Analysis

**Figure 7:**
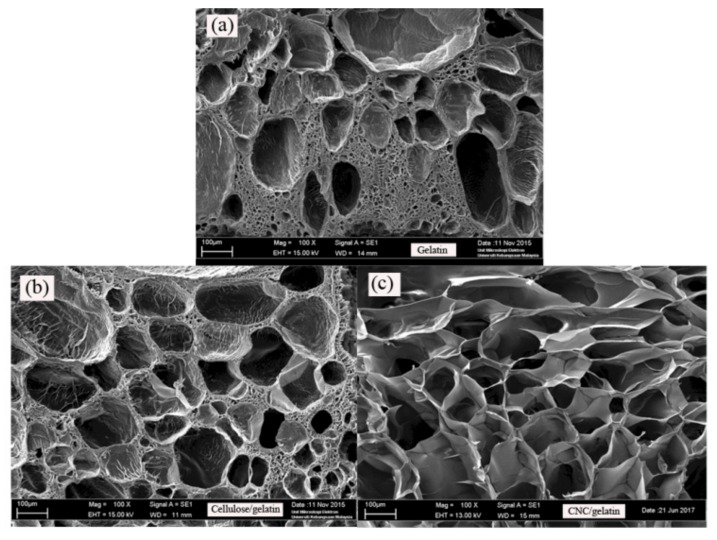
Micrographs of swollen (**a**) gelatin hydrogel; (**b**) hydrogel A; and (**c**) hydrogel B.

Figure 7 shows SEM images of pure gelatin, and A and B hydrogels, revealing the morphological differences between swollen and freeze-dried samples and demonstrating that the incorporation of cellulosic fibers generally improved the hydrogel pore structure by affecting pore arrangement, regularity, and rigidity. Thus, cellulosic fibers were concluded to promote the formation of stable pores by supporting the structure of gelatin. 

Hydrogel B had more regular and rigidly shaped pores than hydrogel A, which, again, was ascribed to the small size and, hence, better dispersion of CNCs. The thus-produced uniform and structured pore distribution allowed the penetration of water molecules to occur without the destruction of the gel structure-maintaining interactions. Conversely, the irregular pore shape and arrangement observed for hydrogel A was due to the increased dimensions of gelatin gel-filled spaces between cellulose fibers, with the agglomeration of cellulosic fibers resulting in compact pore formation. 

Water-absorption speed and water uptake are closely related to the size, structure, and distribution of hydrogel pores. The results of SEM imaging were in good agreement with those obtained by swelling degree analysis (Figure 6), being potentially useful for improving the water-absorption capacity/rate of hydrogels [12]. 

### 3.7. In Vitro Drug Profiling

**Figure 8:**
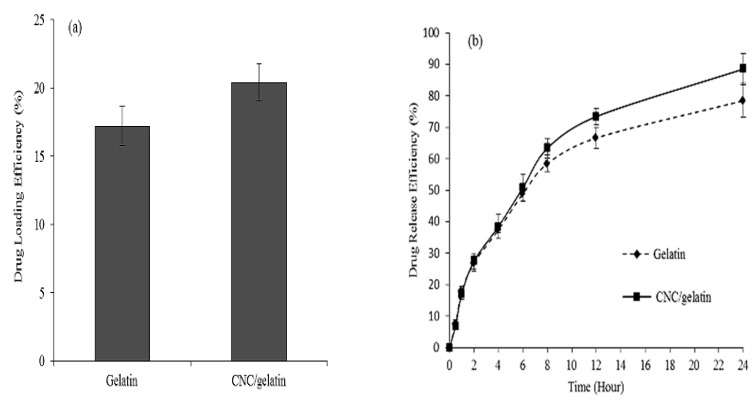
Riboflavin/drug (**a**) loading and (**b**) release efficiencies of hydrogels.

The potential of hydrogels as drug-delivery systems is commonly studied based on in vitro drug loading and release. Herein, only a CNC/gelatin hydrogel was subjected to the above test due to exhibiting remarkable swelling ability, pore structure, and rheological properties, with the pure gelatin hydrogel used as a control. Figure 8 shows the drug-loading and release efficiencies of gelatin and CNC/gelatin hydrogel over 24 h, revealing that these efficiencies increased with time in both cases, which indicated that the above hydrogel was potentially suited for use in drug-delivery systems. 

Figure 8a shows that a riboflavin loading of 20% was achieved for the CNC/gelatin hydrogel, whereas a value of only 17% was observed for the pure-gelatin hydrogel, which indicated the higher drug-loading efficiency of the former. Obviously, the observed trend was coherent with that of the swelling ratio pattern [21]. When hydrogels were immersed in drug solution, penetration of the solvent through their pores induced swelling and thus allowed more drug molecules to be incorporated. Hence, it was concluded that drug-loading efficiency depends on swelling behavior, e.g., the CNC/gelatin hydrogel was more rigid and exhibited a higher storage modulus and more rigid pores [12] than the gelatin hydrogel, consequently being a better drug carrier due to allowing more drug molecules to diffuse within a given time period.

The efficiency of drug release from the CNC/gelatin hydrogel exceeded that of the gelatin hydrogel, as shown in Figure 8b, i.e., 73% and 66% of riboflavin was released from the above systems within 12 h, respectively, in agreement with the swelling ratio pattern in Figure 6. Herein, drug release was controlled by a solvent-activation or swelling-controlled mechanism, that is, riboflavin trapped within the polymer matrix was released by polymer swelling due to the osmotic effect. Specifically, the osmotic driving force initiated the external water/solvent to enter the drug delivery system and drive out the drug [28]. 

Based on Figure 7, the increase in swelling ratio was altered by the formation of voids in hydrogels, with the CNC hybridization-promoted formation of regular and rigid voids leading to more efficient drug release. CNCs also acted as strong reinforcing materials controlling the pore structure of gelatin hydrogels, maintaining their rigidity and helping to sustain void formation under the action of the osmotic driving force, thus allowing drug molecules to easily penetrate pores and exit into the buffer solution.

The kinetic mechanism of drug release has been studied by using a mathematical analysis approached on experimental data. An interesting model developed by Peppas-Sahlin [29], which quantifies and materializes the amount of drug released by Fickian diffusion and by polymer relaxation, was chosen. Drug-release data have been treated by Peppas-Sahlin equation:

(3) $$\frac{M_{t}}{M_{\infty }}\;=\;K_{1}t^{n}\;+\;K_{2}t^{2n}$$

where *M*_t_/*M*_∞_ is the fraction of drug release at a time, *t* and *n* are diffusional exponents, while *K*_1_ and *K*_2_ are kinetic constants. The values of *K*_1_ indicate the contribution of diffusion (Fickian) and the value of *K*_2_ is associated with polymer relaxation, respectively. The results of the treated experimental data by the mathematical analysis are recorded in Table 2. The Peppas-Sahlin model indicates that, when the value of exponent *n* is 0.45, drug release follows a Fickian-type diffusion mechanism. An abnormal diffusion or non-Fickian diffusion occurs when the value of *n* is >0.45 but smaller than 1. Meanwhile, when *n* = 1, the release kinetic system is known as zero order (transport Case II) [30]. Based on Table 2, the diffusional exponent, *n* values for this drug kinetic study are between 0.45 and 1, suggesting an abnormal or non-Fickian diffusion. Therefore, diffusion rate depends on the drug-concentration gradient [21]. 

The regression coefficient (*R*^2^) is the most common method to assess the fitting of a model equation. It is notable that the *R*^2^ of the drug-release data is higher than 0.90, which is high enough to be evaluated with the Peppas-Sahlin model. The value of diffusion constant *K*_1_ is remarkably higher than the value of relaxation constant *K*_2_ for both hydrogels, which indicates the predominance of the swelling mechanism over the erosion mechanism combined with the high solubility of drugs [21].

## 4. Conclusions

Herein, we describe the preparation of crosslinked gelatin/cellulose-containing hydrogels, utilizing gamma irradiation for chemical reagent-free crosslinking and employing cellulose fibers isolated from rice husks and CNCs as hydrogel-reinforcing agents. Compared to that of regular fibrous cellulose, incorporation of CNCs resulted in higher swelling ability, better dynamic mechanical properties, and enhanced drug uptake/release performance, i.e., the size of included cellulose markedly affected the performance of crosslinked hydrogels, in agreement with our original hypothesis. CNC/gelatin hydrogels produced are potentially suitable for drug-delivery applications, suggesting a future extended direction of the present method.

## Figures and Tables

**Table 1:** CrI values for cellulose and CNC XRD peaks.

Fiber	CrI (%) at 2*θ*		
	16°	22°	35°
Cellulose	42	72	22
CNCs	54	74	37

**Table 2:** Fitting paramaters obtained from the Peppas-Sahlin equation.

Hydrogels	Kinetic Constant (*K*_1_)	Kinetic Constant (*K*_2_)	Regression Coefficient (*R*^2^)	*n*
Gelatin	95.85	0.45	0.97	0.68
CNC/Gelatin	100.62	0.48	0.97	0.75
